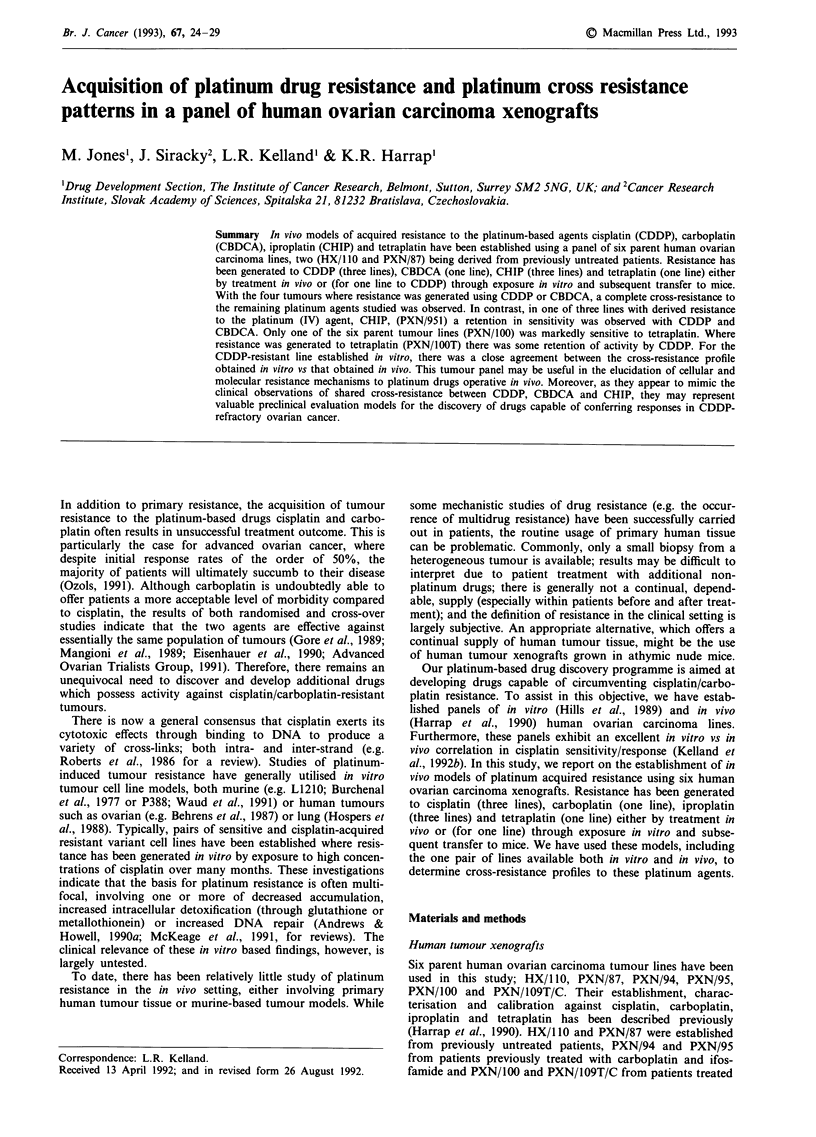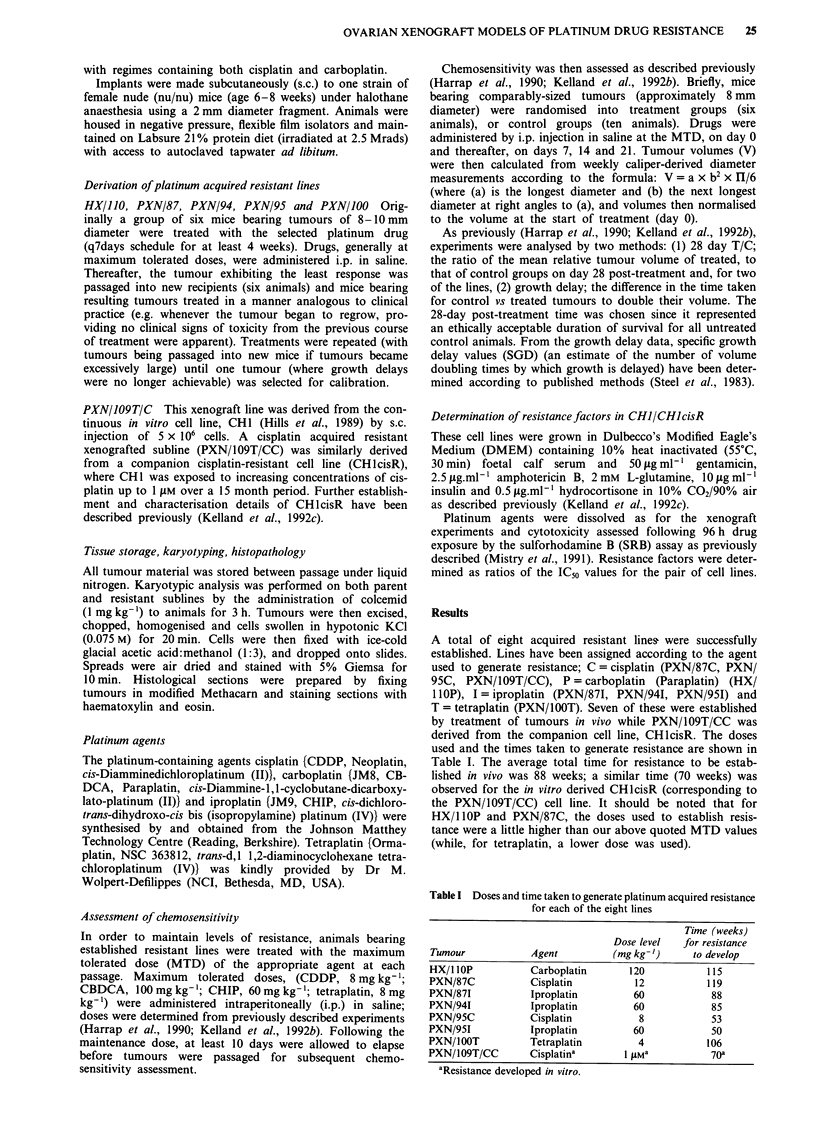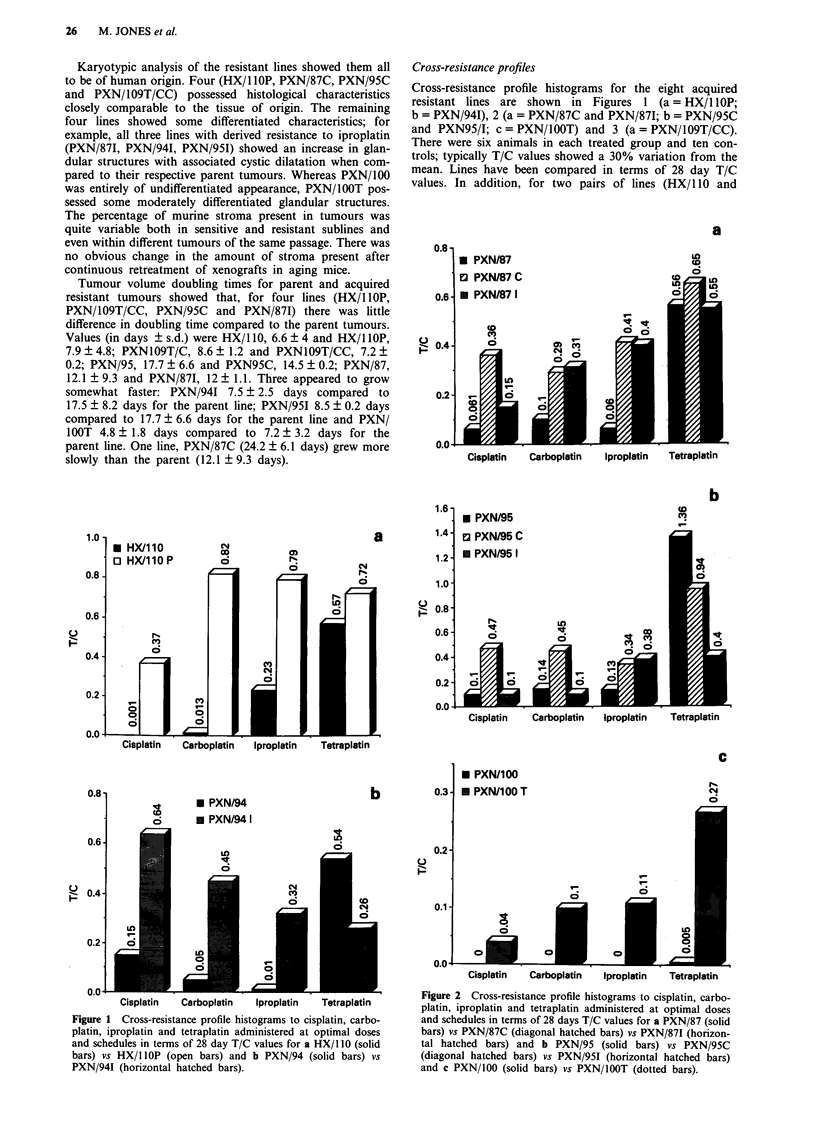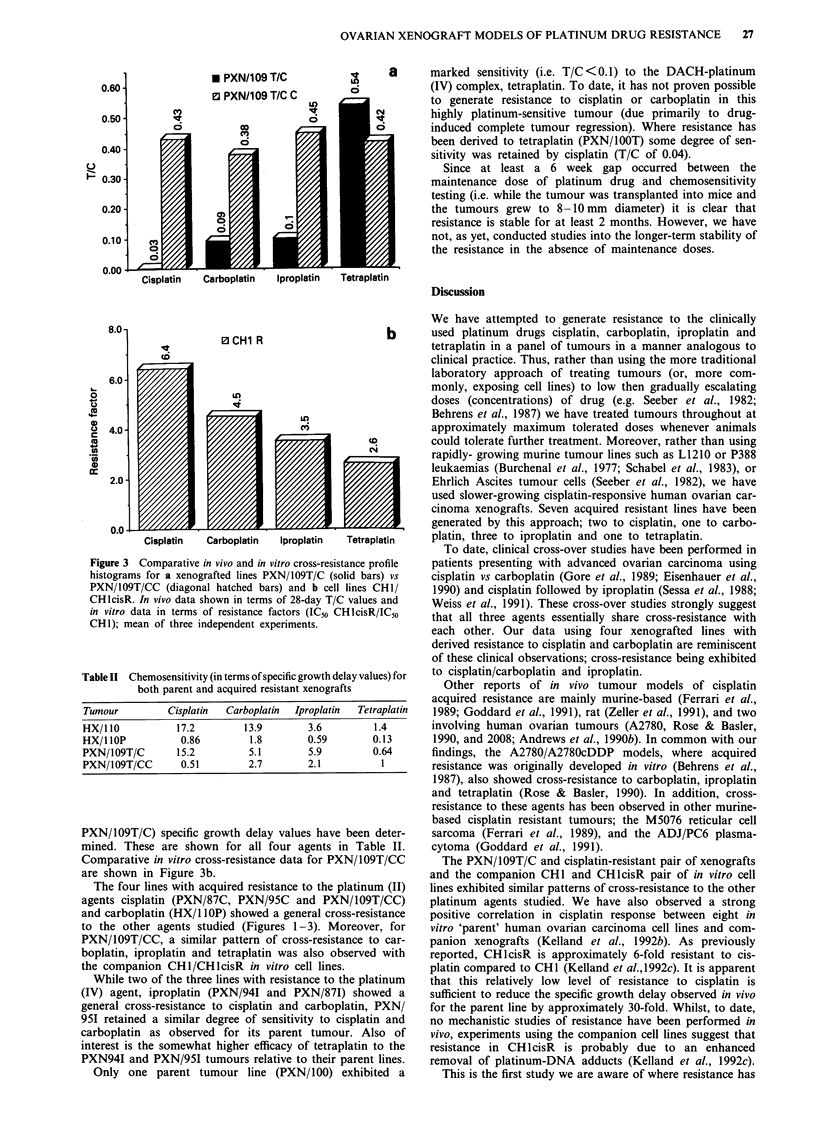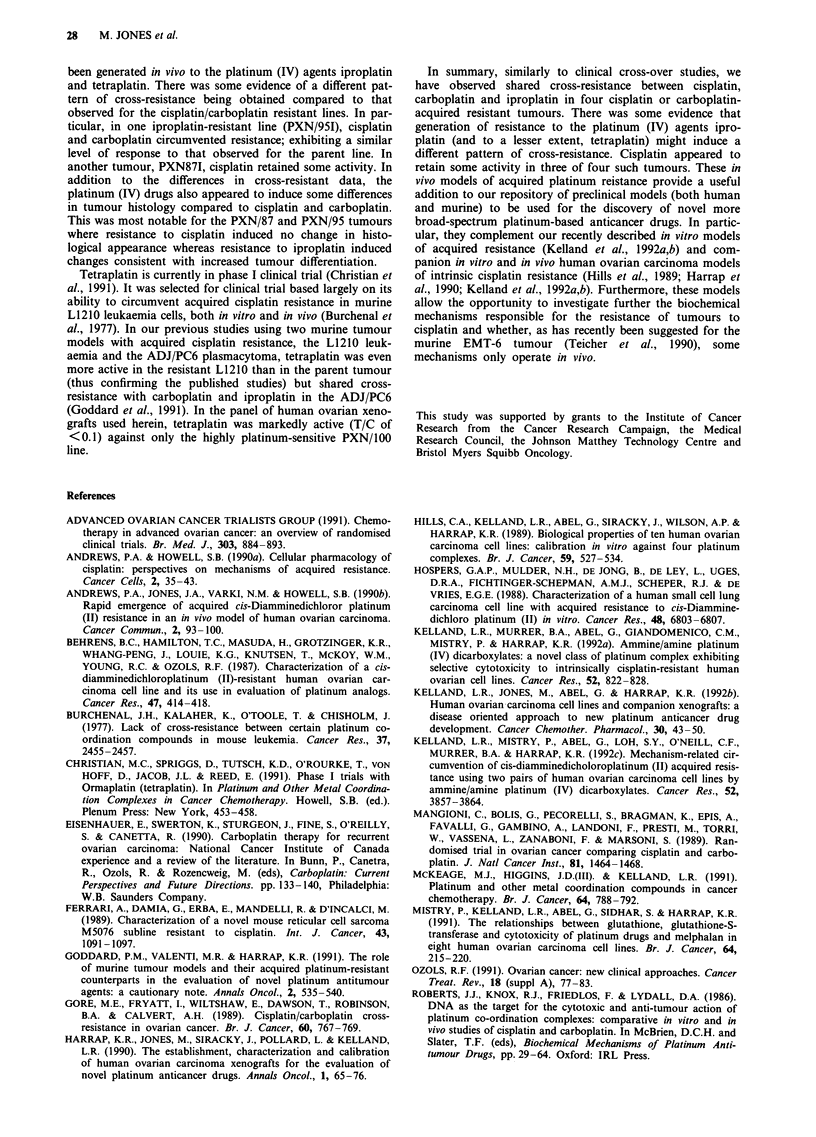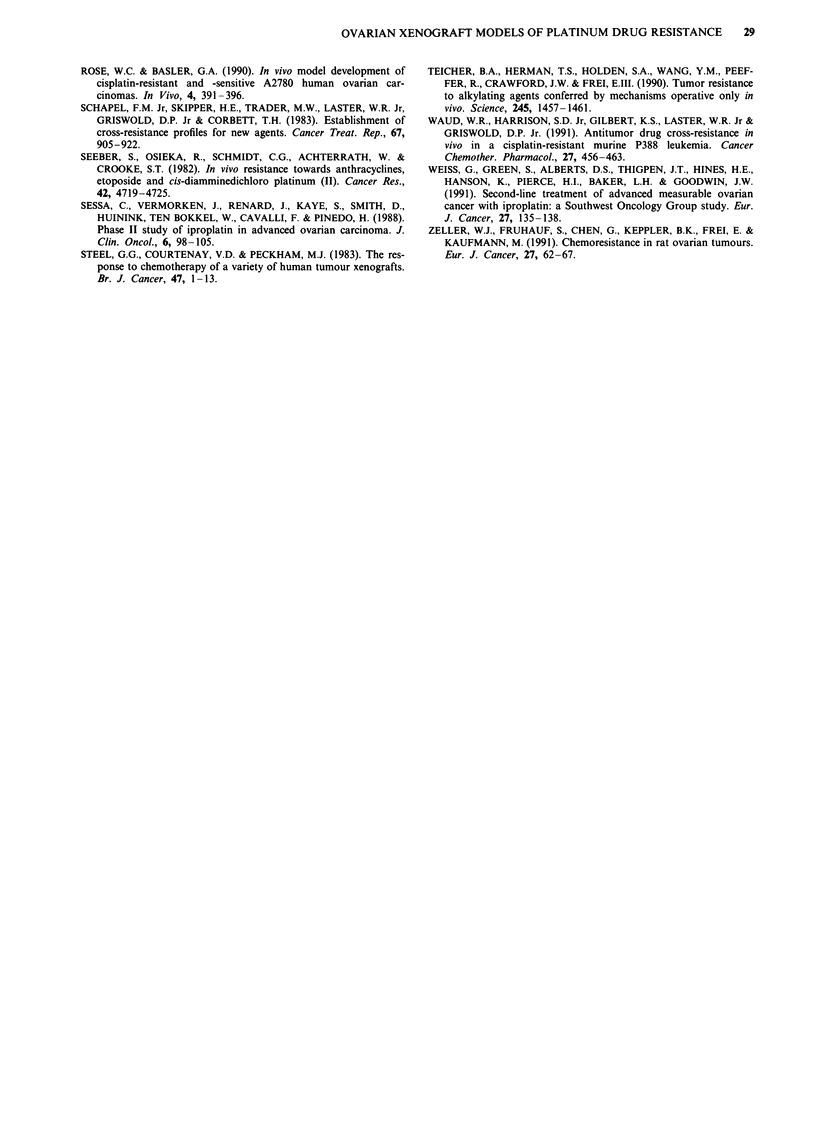# Acquisition of platinum drug resistance and platinum cross resistance patterns in a panel of human ovarian carcinoma xenografts.

**DOI:** 10.1038/bjc.1993.5

**Published:** 1993-01

**Authors:** M. Jones, J. Siracky, L. R. Kelland, K. R. Harrap

**Affiliations:** Drug Development Section, Institute of Cancer Research, Sutton, Surrey, UK.

## Abstract

In vivo models of acquired resistance to the platinum-based agents cisplatin (CDDP), carboplatin (CBDCA), iproplatin (CHIP) and tetraplatin have been established using a panel of six parent human ovarian carcinoma lines, two (HX/110 and PXN/87) being derived from previously untreated patients. Resistance has been generated to CDDP (three lines), CBDCA (one line), CHIP (three lines) and tetraplatin (one line) either by treatment in vivo or (for one line to CDDP) through exposure in vitro and subsequent transfer to mice. With the four tumours where resistance was generated using CDDP or CBDCA, a complete cross-resistance to the remaining platinum agents studied was observed. In contrast, in one of three lines with derived resistance to the platinum (IV) agent, CHIP, (PXN/951) a retention in sensitivity was observed with CDDP and CBDCA. Only one of the six parent tumour lines (PXN/100) was markedly sensitive to tetraplatin. Where resistance was generated to tetraplatin (PXN/100T) there was some retention of activity by CDDP. For the CDDP-resistant line established in vitro, there was a close agreement between the cross-resistance profile obtained in vitro vs that obtained in vivo. This tumour panel may be useful in the elucidation of cellular and molecular resistance mechanisms to platinum drugs operative in vivo. Moreover, as they appear to mimic the clinical observations of shared cross-resistance between CDDP, CBDCA and CHIP, they may represent valuable preclinical evaluation models for the discovery of drugs capable of conferring responses in CDDP-refractory ovarian cancer.


Br. J. Cancer (1993), 67, 24-29 © Macmillan Press Ltd., 1993

### Acquisition of platinum drug resistance and platinum cross resistance patterns in a panel of human ovarian carcinoma xenografts

M. Jones[1], J. Siracky[2], L.R. Kelland[1] & K.R. Harrap[1]

[1]*Drug Development Section, The Institute of Cancer Research, Belmont, Sutton, Surrey SM2 5NG, UK; and* [2]*Cancer Research Institute, Slovak Academy of Sciences, Spitalska 21, 81232 Bratislava, Czechoslovakia.*

**Summary** *In vivo* models of acquired resistance to the platinum-based agents cisplatin (CDDP), carboplatin (CBDCA), iproplatin (CHIP) and tetraplatin have been established using a panel of six parent human ovarian carcinoma lines, two (HX/110 and PXN/87) being derived from previously untreated patients. Resistance has been generated to CDDP (three lines), CBDCA (one line), CHIP (three lines) and tetraplatin (one line) either by treatment *in vivo* or (for one line to CDDP) through exposure *in vitro* and subsequent transfer to mice. With the four tumours where resistance was generated using CDDP or CBDCA, a complete cross-resistance to the remaining platinum agents studied was observed. In contrast, in one of three lines with derived resistance to the platinum (IV) agent, CHIP, (PXN/951) a retention in sensitivity was observed with CDDP and CBDCA. Only one of the six parent tumour lines (PXN/100) was markedly sensitive to tetraplatin. Where resistance was generated to tetraplatin (PXN/100T) there was some retention of activity by CDDP. For the CDDP-resistant line established *in vitro*, there was a close agreement between the cross-resistance profile obtained *in vitro vs* that obtained *in vivo*. This tumour panel may be useful in the elucidation of cellular and molecular resistance mechanisms to platinum drugs operative *in vivo*. Moreover, as they appear to mimic the clinical observations of shared cross-resistance between CDDP, CBDCA and CHIP, they may represent valuable preclinical evaluation models for the discovery of drugs capable of conferring responses in CDDP-refractory ovarian cancer.

In addition to primary resistance, the acquisition of tumour resistance to the platinum-based drugs cisplatin and carboplatin often results in unsuccessful treatment outcome. This is particularly the case for advanced ovarian cancer, where despite initial response rates of the order of 50%, the majority of patients will ultimately succumb to their disease (Ozols, 1991). Although carboplatin is undoubtedly able to offer patients a more acceptable level of morbidity compared to cisplatin, the results of both randomised and cross-over studies indicate that the two agents are effective against essentially the same population of tumours (Gore *et al.*, 1989; Mangioni *et al.*, 1989; Eisenhauer *et al.*, 1990; Advanced Ovarian Trialists Group, 1991). Therefore, there remains an unequivocal need to discover and develop additional drugs which possess activity against cisplatin/carboplatin-resistant tumours.

There is now a general consensus that cisplatin exerts its cytotoxic effects through binding to DNA to produce a variety of cross-links; both intra- and inter-strand (e.g. Roberts *et al.*, 1986 for a review). Studies of platinum-induced tumour resistance have generally utilised *in vitro* tumour cell line models, both murine (e.g. L1210; Burchenal *et al.*, 1977 or P388; Waud *et al.*, 1991) or human tumours such as ovarian (e.g. Behrens *et al.*, 1987) or lung (Hospers *et al.*, 1988). Typically, pairs of sensitive and cisplatin-acquired resistant variant cell lines have been established where resistance has been generated *in vitro* by exposure to high concentrations of cisplatin over many months. These investigations indicate that the basis for platinum resistance is often multifocal, involving one or more of decreased accumulation, increased intracellular detoxification (through glutathione or metallothionein) or increased DNA repair (Andrews & Howell, 1990*a*; McKeage *et al.*, 1991, for reviews). The clinical relevance of these *in vitro* based findings, however, is largely untested.

To date, there has been relatively little study of platinum resistance in the *in vivo* setting, either involving primary human tumour tissue or murine-based tumour models. While some mechanistic studies of drug resistance (e.g. the occurrence of multidrug resistance) have been successfully carried out in patients, the routine usage of primary human tissue can be problematic. Commonly, only a small biopsy from a heterogeneous tumour is available; results may be difficult to interpret due to patient treatment with additional non-platinum drugs; there is generally not a continual, dependable, supply (especially within patients before and after treatment); and the definition of resistance in the clinical setting is largely subjective. An appropriate alternative, which offers a continual supply of human tumour tissue, might be the use of human tumour xenografts grown in athymic nude mice.

Our platinum-based drug discovery programme is aimed at developing drugs capable of circumventing cisplatin/carboplatin resistance. To assist in this objective, we have established panels of *in vitro* (Hills *et al.*, 1989) and *in vivo* (Harrap *et al.*, 1990) human ovarian carcinoma lines. Furthermore, these panels exhibit an excellent *in vitro vs in vivo* correlation in cisplatin sensitivity/response (Kelland *et al.*, 1992*b*). In this study, we report on the establishment of *in vivo* models of platinum acquired resistance using six human ovarian carcinoma xenografts. Resistance has been generated to cisplatin (three lines), carboplatin (one line), iproplatin (three lines) and tetraplatin (one line) either by treatment *in vivo* or (for one line) through exposure *in vitro* and subsequent transfer to mice. We have used these models, including the one pair of lines available both *in vitro* and *in vivo*, to determine cross-resistance profiles to these platinum agents.

### Materials and methods

### *Human tumour xenografts*

Six parent human ovarian carcinoma tumour lines have been used in this study; HX/110, PXN/87, PXN/94, PXN/95, PXN/100 and PXN/109T/C. Their establishment, characterisation and calibration against cisplatin, carboplatin, iproplatin and tetraplatin has been described previously (Harrap *et al.*, 1990). HX/110 and PXN/87 were established from previously untreated patients, PXN/94 and PXN/95 from patients previously treated with carboplatin and ifosfamide and PXN/100 and PXN/109T/C from patients treated

Correspondence: L.R. Kelland.
Received 13 April 1992; and in revised form 26 August 1992.

with regimes containing both cisplatin and carboplatin.

Implants were made subcutaneously (s.c.) to one strain of female nude (nu/nu) mice (age 6–8 weeks) under halothane anaesthesia using a 2 mm diameter fragment. Animals were housed in negative pressure, flexible film isolators and maintained on Labsure 21% protein diet (irradiated at 2.5 Mrads) with access to autoclaved tapwater *ad libitum*.

### *Derivation of platinum acquired resistant lines*

*HX/110, PXN/87, PXN/94, PXN/95 and PXN/100* Originally a group of six mice bearing tumours of 8–10 mm diameter were treated with the selected platinum drug (q7days schedule for at least 4 weeks). Drugs, generally at maximum tolerated doses, were administered i.p. in saline. Thereafter, the tumour exhibiting the least response was passaged into new recipients (six animals) and mice bearing resulting tumours treated in a manner analogous to clinical practice (e.g. whenever the tumour began to regrow, providing no clinical signs of toxicity from the previous course of treatment were apparent). Treatments were repeated (with tumours being passaged into new mice if tumours became excessively large) until one tumour (where growth delays were no longer achievable) was selected for calibration.

*PXN/109T/C* This xenograft line was derived from the continuous *in vitro* cell line, CH1 (Hills *et al.*, 1989) by s.c. injection of $5 \times 10^6$ cells. A cisplatin acquired resistant xenografted subline (PXN/109T/CC) was similarly derived from a companion cisplatin-resistant cell line (CH1cisR), where CH1 was exposed to increasing concentrations of cisplatin up to 1 μM over a 15 month period. Further establishment and characterisation details of CH1cisR have been described previously (Kelland *et al.*, 1992*c*).

### *Tissue storage, karyotyping, histopathology*

All tumour material was stored between passage under liquid nitrogen. Karyotypic analysis was performed on both parent and resistant sublines by the administration of colcemid (1 mg $kg^{-1}$) to animals for 3 h. Tumours were then excised, chopped, homogenised and cells swollen in hypotonic KCl (0.075 M) for 20 min. Cells were then fixed with ice-cold glacial acetic acid:methanol (1:3), and dropped onto slides. Spreads were air dried and stained with 5% Giemsa for 10 min. Histological sections were prepared by fixing tumours in modified Methacarn and staining sections with haematoxylin and eosin.

### *Platinum agents*

The platinum-containing agents cisplatin {CDDP, Neoplatin, *cis*-Diamminedichloroplatinum (II)}, carboplatin {JM8, CBDCA, Paraplatin, *cis*-Diammine-1,1-cyclobutane-dicarboxylato-platinum (II)} and iproplatin {JM9, CHIP, *cis*-dichloro-*trans*-dihydroxo-*cis* bis (isopropylamine) platinum (IV)} were synthesised by and obtained from the Johnson Matthey Technology Centre (Reading, Berkshire). Tetraplatin {Ormaplatin, NSC 363812, *trans*-d,1 1,2-diaminocyclohexane tetrachloroplatinum (IV)} was kindly provided by Dr M. Wolpert-Defilippes (NCI, Bethesda, MD, USA).

### *Assessment of chemosensitivity*

In order to maintain levels of resistance, animals bearing established resistant lines were treated with the maximum tolerated dose (MTD) of the appropriate agent at each passage. Maximum tolerated doses, (CDDP, 8 mg $kg^{-1}$; CBDCA, 100 mg $kg^{-1}$; CHIP, 60 mg $kg^{-1}$; tetraplatin, 8 mg $kg^{-1}$) were administered intraperitoneally (i.p.) in saline; doses were determined from previously described experiments (Harrap *et al.*, 1990; Kelland *et al.*, 1992*b*). Following the maintenance dose, at least 10 days were allowed to elapse before tumours were passaged for subsequent chemosensitivity assessment.

Chemosensitivity was then assessed as described previously (Harrap *et al.*, 1990; Kelland *et al.*, 1992*b*). Briefly, mice bearing comparably-sized tumours (approximately 8 mm diameter) were randomised into treatment groups (six animals), or control groups (ten animals). Drugs were administered by i.p. injection in saline at the MTD, on day 0 and thereafter, on days 7, 14 and 21. Tumour volumes (V) were then calculated from weekly caliper-derived diameter measurements according to the formula: $V = a \times b^2 \times \Pi/6$ (where (a) is the longest diameter and (b) the next longest diameter at right angles to (a), and volumes then normalised to the volume at the start of treatment (day 0).

As previously (Harrap *et al.*, 1990; Kelland *et al.*, 1992*b*), experiments were analysed by two methods: (1) 28 day T/C; the ratio of the mean relative tumour volume of treated, to that of control groups on day 28 post-treatment and, for two of the lines, (2) growth delay; the difference in the time taken for control *vs* treated tumours to double their volume. The 28-day post-treatment time was chosen since it represented an ethically acceptable duration of survival for all untreated control animals. From the growth delay data, specific growth delay values (SGD) (an estimate of the number of volume doubling times by which growth is delayed) have been determined according to published methods (Steel *et al.*, 1983).

### *Determination of resistance factors in CH1/CH1cisR*

These cell lines were grown in Dulbecco's Modified Eagle's Medium (DMEM) containing 10% heat inactivated (55°C, 30 min) foetal calf serum and 50 μg $ml^{-1}$ gentamicin, 2.5 μg.$ml^{-1}$ amphotericin B, 2 mM L-glutamine, 10 μg $ml^{-1}$ insulin and 0.5 μg.$ml^{-1}$ hydrocortisone in 10% $CO_2$/90% air as described previously (Kelland *et al.*, 1992*c*).

Platinum agents were dissolved as for the xenograft experiments and cytotoxicity assessed following 96 h drug exposure by the sulforhodamine B (SRB) assay as previously described (Mistry *et al.*, 1991). Resistance factors were determined as ratios of the $IC_{50}$ values for the pair of cell lines.

### Results

A total of eight acquired resistant lines were successfully established. Lines have been assigned according to the agent used to generate resistance; C = cisplatin (PXN/87C, PXN/95C, PXN/109T/CC), P = carboplatin (Paraplatin) (HX/110P), I = iproplatin (PXN/87I, PXN/94I, PXN/95I) and T = tetraplatin (PXN/100T). Seven of these were established by treatment of tumours *in vivo* while PXN/109T/CC was derived from the companion cell line, CH1cisR. The doses used and the times taken to generate resistance are shown in Table I. The average total time for resistance to be established *in vivo* was 88 weeks; a similar time (70 weeks) was observed for the *in vitro* derived CH1cisR (corresponding to the PXN/109T/CC) cell line. It should be noted that for HX/110P and PXN/87C, the doses used to establish resistance were a little higher than our above quoted MTD values (while, for tetraplatin, a lower dose was used).

**Table I** Doses and time taken to generate platinum acquired resistance for each of the eight lines

| *Tumour* | *Agent* | *Dose level ($mg\ kg^{-1}$)* | *Time (weeks) for resistance to develop* |
|---|---|---|---|
| HX/110P | Carboplatin | 120 | 115 |
| PXN/87C | Cisplatin | 12 | 119 |
| PXN/87I | Iproplatin | 60 | 88 |
| PXN/94I | Iproplatin | 60 | 85 |
| PXN/95C | Cisplatin | 8 | 53 |
| PXN/95I | Iproplatin | 60 | 50 |
| PXN/100T | Tetraplatin | 4 | 106 |
| PXN/109T/CC | Cisplatin[a] | 1 μM[a] | 70[a] |

[a]Resistance developed *in vitro*.

Karyotypic analysis of the resistant lines showed them all to be of human origin. Four (HX/110P, PXN/87C, PXN/95C and PXN/109T/CC) possessed histological characteristics closely comparable to the tissue of origin. The remaining four lines showed some differentiated characteristics; for example, all three lines with derived resistance to iproplatin (PXN/87I, PXN/94I, PXN/95I) showed an increase in glandular structures with associated cystic dilatation when compared to their respective parent tumours. Whereas PXN/100 was entirely of undifferentiated appearance, PXN/100T possessed some moderately differentiated glandular structures. The percentage of murine stroma present in tumours was quite variable both in sensitive and resistant sublines and even within different tumours of the same passage. There was no obvious change in the amount of stroma present after continuous retreatment of xenografts in aging mice.

Tumour volume doubling times for parent and acquired resistant tumours showed that, for four lines (HX/110P, PXN/109T/CC, PXN/95C and PXN/87I) there was little difference in doubling time compared to the parent tumours. Values (in days ± s.d.) were HX/110, 6.6 ± 4 and HX/110P, 7.9 ± 4.8; PXN109T/C, 8.6 ± 1.2 and PXN109T/CC, 7.2 ± 0.2; PXN/95, 17.7 ± 6.6 and PXN95C, 14.5 ± 0.2; PXN/87, 12.1 ± 9.3 and PXN/87I, 12 ± 1.1. Three appeared to grow somewhat faster: PXN/94I 7.5 ± 2.5 days compared to 17.5 ± 8.2 days for the parent line; PXN/95I 8.5 ± 0.2 days compared to 17.7 ± 6.6 days for the parent line and PXN/100T 4.8 ± 1.8 days compared to 7.2 ± 3.2 days for the parent line. One line, PXN/87C (24.2 ± 6.1 days) grew more slowly than the parent (12.1 ± 9.3 days).

*Cross-resistance profiles*

Cross-resistance profile histograms for the eight acquired resistant lines are shown in Figures 1 (a = HX/110P; b = PXN/94I), 2 (a = PXN/87C and PXN/87I; b = PXN/95C and PXN95/I; c = PXN/100T) and 3 (a = PXN/109T/CC). There were six animals in each treated group and ten controls; typically T/C values showed a 30% variation from the mean. Lines have been compared in terms of 28 day T/C values. In addition, for two pairs of lines (HX/110 and

**Figure 1** Cross-resistance profile histograms to cisplatin, carboplatin, iproplatin and tetraplatin administered at optimal doses and schedules in terms of 28 day T/C values for **a** HX/110 (solid bars) *vs* HX/110P (open bars) and **b** PXN/94 (solid bars) *vs* PXN/94I (horizontal hatched bars).

**Figure 2** Cross-resistance profile histograms to cisplatin, carboplatin, iproplatin and tetraplatin administered at optimal doses and schedules in terms of 28 days T/C values for **a** PXN/87 (solid bars) *vs* PXN/87C (diagonal hatched bars) *vs* PXN/87I (horizontal hatched bars) and **b** PXN/95 (solid bars) *vs* PXN/95C (diagonal hatched bars) *vs* PXN/95I (horizontal hatched bars) and **c** PXN/100 (solid bars) *vs* PXN/100T (dotted bars).

**Figure 3** Comparative *in vivo* and *in vitro* cross-resistance profile histograms for **a** xenografted lines PXN/109T/C (solid bars) *vs* PXN/109T/CC (diagonal hatched bars) and **b** cell lines CH1/CH1cisR. *In vivo* data shown in terms of 28-day T/C values and *in vitro* data in terms of resistance factors ($IC_{50}$ CH1cisR/$IC_{50}$ CH1); mean of three independent experiments.

**Table II** Chemosensitivity (in terms of specific growth delay values) for both parent and acquired resistant xenografts

| *Tumour* | *Cisplatin* | *Carboplatin* | *Iproplatin* | *Tetraplatin* |
|---|---|---|---|---|
| HX/110 | 17.2 | 13.9 | 3.6 | 1.4 |
| HX/110P | 0.86 | 1.8 | 0.59 | 0.13 |
| PXN/109T/C | 15.2 | 5.1 | 5.9 | 0.64 |
| PXN/109T/CC | 0.51 | 2.7 | 2.1 | 1 |

PXN/109T/C) specific growth delay values have been determined. These are shown for all four agents in Table II. Comparative *in vitro* cross-resistance data for PXN/109T/CC are shown in Figure 3b.

The four lines with acquired resistance to the platinum (II) agents cisplatin (PXN/87C, PXN/95C and PXN/109T/CC) and carboplatin (HX/110P) showed a general cross-resistance to the other agents studied (Figures 1–3). Moreover, for PXN/109T/CC, a similar pattern of cross-resistance to carboplatin, iproplatin and tetraplatin was also observed with the companion CH1/CH1cisR *in vitro* cell lines.

While two of the three lines with resistance to the platinum (IV) agent, iproplatin (PXN/94I and PXN/87I) showed a general cross-resistance to cisplatin and carboplatin, PXN/95I retained a similar degree of sensitivity to cisplatin and carboplatin as observed for its parent tumour. Also of interest is the somewhat higher efficacy of tetraplatin to the PXN94I and PXN/95I tumours relative to their parent lines.

Only one parent tumour line (PXN/100) exhibited a marked sensitivity (i.e. T/C<0.1) to the DACH-platinum (IV) complex, tetraplatin. To date, it has not proven possible to generate resistance to cisplatin or carboplatin in this highly platinum-sensitive tumour (due primarily to drug-induced complete tumour regression). Where resistance has been derived to tetraplatin (PXN/100T) some degree of sensitivity was retained by cisplatin (T/C of 0.04).

Since at least a 6 week gap occurred between the maintenance dose of platinum drug and chemosensitivity testing (i.e. while the tumour was transplanted into mice and the tumours grew to 8–10 mm diameter) it is clear that resistance is stable for at least 2 months. However, we have not, as yet, conducted studies into the longer-term stability of the resistance in the absence of maintenance doses.

### Discussion

We have attempted to generate resistance to the clinically used platinum drugs cisplatin, carboplatin, iproplatin and tetraplatin in a panel of tumours in a manner analogous to clinical practice. Thus, rather than using the more traditional laboratory approach of treating tumours (or, more commonly, exposing cell lines) to low then gradually escalating doses (concentrations) of drug (e.g. Seeber *et al.*, 1982; Behrens *et al.*, 1987) we have treated tumours throughout at approximately maximum tolerated doses whenever animals could tolerate further treatment. Moreover, rather than using rapidly- growing murine tumour lines such as L1210 or P388 leukaemias (Burchenal *et al.*, 1977; Schabel *et al.*, 1983), or Ehrlich Ascites tumour cells (Seeber *et al.*, 1982), we have used slower-growing cisplatin-responsive human ovarian carcinoma xenografts. Seven acquired resistant lines have been generated by this approach; two to cisplatin, one to carboplatin, three to iproplatin and one to tetraplatin.

To date, clinical cross-over studies have been performed in patients presenting with advanced ovarian carcinoma using cisplatin *vs* carboplatin (Gore *et al.*, 1989; Eisenhauer *et al.*, 1990) and cisplatin followed by iproplatin (Sessa *et al.*, 1988; Weiss *et al.*, 1991). These cross-over studies strongly suggest that all three agents essentially share cross-resistance with each other. Our data using four xenografted lines with derived resistance to cisplatin and carboplatin are reminiscent of these clinical observations; cross-resistance being exhibited to cisplatin/carboplatin and iproplatin.

Other reports of *in vivo* tumour models of cisplatin acquired resistance are mainly murine-based (Ferrari *et al.*, 1989; Goddard *et al.*, 1991), rat (Zeller *et al.*, 1991), and two involving human ovarian tumours (A2780, Rose & Basler, 1990, and 2008; Andrews *et al.*, 1990*b*). In common with our findings, the A2780/A2780cDDP models, where acquired resistance was originally developed *in vitro* (Behrens *et al.*, 1987), also showed cross-resistance to carboplatin, iproplatin and tetraplatin (Rose & Basler, 1990). In addition, cross-resistance to these agents has been observed in other murine-based cisplatin resistant tumours; the M5076 reticular cell sarcoma (Ferrari *et al.*, 1989), and the ADJ/PC6 plasmacytoma (Goddard *et al.*, 1991).

The PXN/109T/C and cisplatin-resistant pair of xenografts and the companion CH1 and CH1cisR pair of *in vitro* cell lines exhibited similar patterns of cross-resistance to the other platinum agents studied. We have also observed a strong positive correlation in cisplatin response between eight *in vitro* 'parent' human ovarian carcinoma cell lines and companion xenografts (Kelland *et al.*, 1992*b*). As previously reported, CH1cisR is approximately 6-fold resistant to cisplatin compared to CH1 (Kelland *et al.*,1992*c*). It is apparent that this relatively low level of resistance to cisplatin is sufficient to reduce the specific growth delay observed *in vivo* for the parent line by approximately 30-fold. Whilst, to date, no mechanistic studies of resistance have been performed *in vivo*, experiments using the companion cell lines suggest that resistance in CH1cisR is probably due to an enhanced removal of platinum-DNA adducts (Kelland *et al.*, 1992*c*).

This is the first study we are aware of where resistance has been generated *in vivo* to the platinum (IV) agents iproplatin and tetraplatin. There was some evidence of a different pattern of cross-resistance being obtained compared to that observed for the cisplatin/carboplatin resistant lines. In particular, in one iproplatin-resistant line (PXN/95I), cisplatin and carboplatin circumvented resistance; exhibiting a similar level of response to that observed for the parent line. In another tumour, PXN87I, cisplatin retained some activity. In addition to the differences in cross-resistant data, the platinum (IV) drugs also appeared to induce some differences in tumour histology compared to cisplatin and carboplatin. This was most notable for the PXN/87 and PXN/95 tumours where resistance to cisplatin induced no change in histological appearance whereas resistance to iproplatin induced changes consistent with increased tumour differentiation.

Tetraplatin is currently in phase I clinical trial (Christian *et al.*, 1991). It was selected for clinical trial based largely on its ability to circumvent acquired cisplatin resistance in murine L1210 leukaemia cells, both *in vitro* and *in vivo* (Burchenal *et al.*, 1977). In our previous studies using two murine tumour models with acquired cisplatin resistance, the L1210 leukaemia and the ADJ/PC6 plasmacytoma, tetraplatin was even more active in the resistant L1210 than in the parent tumour (thus confirming the published studies) but shared cross-resistance with carboplatin and iproplatin in the ADJ/PC6 (Goddard *et al.*, 1991). In the panel of human ovarian xenografts used herein, tetraplatin was markedly active (T/C of $<0.1$) against only the highly platinum-sensitive PXN/100 line.

In summary, similarly to clinical cross-over studies, we have observed shared cross-resistance between cisplatin, carboplatin and iproplatin in four cisplatin or carboplatin-acquired resistant tumours. There was some evidence that generation of resistance to the platinum (IV) agents iproplatin (and to a lesser extent, tetraplatin) might induce a different pattern of cross-resistance. Cisplatin appeared to retain some activity in three of four such tumours. These *in vivo* models of acquired platinum reistance provide a useful addition to our repository of preclinical models (both human and murine) to be used for the discovery of novel more broad-spectrum platinum-based anticancer drugs. In particular, they complement our recently described *in vitro* models of acquired resistance (Kelland *et al.*, 1992*a*,*b*) and companion *in vitro* and *in vivo* human ovarian carcinoma models of intrinsic cisplatin resistance (Hills *et al.*, 1989; Harrap *et al.*, 1990; Kelland *et al.*, 1992*a*,*b*). Furthermore, these models allow the opportunity to investigate further the biochemical mechanisms responsible for the resistance of tumours to cisplatin and whether, as has recently been suggested for the murine EMT-6 tumour (Teicher *et al.*, 1990), some mechanisms only operate *in vivo*.

This study was supported by grants to the Institute of Cancer Research from the Cancer Research Campaign, the Medical Research Council, the Johnson Matthey Technology Centre and Bristol Myers Squibb Oncology.

### References

ADVANCED OVARIAN CANCER TRIALISTS GROUP (1991). Chemotherapy in advanced ovarian cancer: an overview of randomised clinical trials. *Br. Med. J.*, **303**, 884–893.

ANDREWS, P.A. & HOWELL, S.B. (1990*a*). Cellular pharmacology of cisplatin: perspectives on mechanisms of acquired resistance. *Cancer Cells*, **2**, 35–43.

ANDREWS, P.A., JONES, J.A., VARKI, N.M. & HOWELL, S.B. (1990*b*). Rapid emergence of acquired *cis*-Diamminedichloror platinum (II) resistance in an *in vivo* model of human ovarian carcinoma. *Cancer Commun.*, **2**, 93–100.

BEHRENS, B.C., HAMILTON, T.C., MASUDA, H., GROTZINGER, K.R., WHANG-PENG, J., LOUIE, K.G., KNUTSEN, T., MCKOY, W.M., YOUNG, R.C. & OZOLS, R.F. (1987). Characterization of a *cis*-diamminedichloroplatinum (II)-resistant human ovarian carcinoma cell line and its use in evaluation of platinum analogs. *Cancer Res.*, **47**, 414–418.

BURCHENAL, J.H., KALAHER, K., O'TOOLE, T. & CHISHOLM, J. (1977). Lack of cross-resistance between certain platinum coordination compounds in mouse leukemia. *Cancer Res.*, **37**, 2455–2457.

CHRISTIAN, M.C., SPRIGGS, D., TUTSCH, K.D., O'ROURKE, T., VON HOFF, D., JACOB, J.L. & REED, E. (1991). Phase I trials with Ormaplatin (tetraplatin). In *Platinum and Other Metal Coordination Complexes in Cancer Chemotherapy*. Howell, S.B. (ed.). Plenum Press: New York, 453–458.

EISENHAUER, E., SWERTON, K., STURGEON, J., FINE, S., O'REILLY, S. & CANETTA, R. (1990). Carboplatin therapy for recurrent ovarian carcinoma: National Cancer Institute of Canada experience and a review of the literature. In Bunn, P., Canetra, R., Ozols, R. & Rozencweig, M. (eds), *Carboplatin: Current Perspectives and Future Directions*. pp. 133–140, Philadelphia: W.B. Saunders Company.

FERRARI, A., DAMIA, G., ERBA, E., MANDELLI, R. & D'INCALCI, M. (1989). Characterization of a novel mouse reticular cell sarcoma M5076 subline resistant to cisplatin. *Int. J. Cancer*, **43**, 1091–1097.

GODDARD, P.M., VALENTI, M.R. & HARRAP, K.R. (1991). The role of murine tumour models and their acquired platinum-resistant counterparts in the evaluation of novel platinum antitumour agents: a cautionary note. *Annals Oncol.*, **2**, 535–540.

GORE, M.E., FRYATT, I., WILTSHAW, E., DAWSON, T., ROBINSON, B.A. & CALVERT, A.H. (1989). Cisplatin/carboplatin cross-resistance in ovarian cancer. *Br. J. Cancer*, **60**, 767–769.

HARRAP, K.R., JONES, M., SIRACKY, J., POLLARD, L. & KELLAND, L.R. (1990). The establishment, characterization and calibration of human ovarian carcinoma xenografts for the evaluation of novel platinum anticancer drugs. *Annals Oncol.*, **1**, 65–76.

HILLS, C.A., KELLAND, L.R., ABEL, G., SIRACKY, J., WILSON, A.P. & HARRAP, K.R. (1989). Biological properties of ten human ovarian carcinoma cell lines: calibration *in vitro* against four platinum complexes. *Br. J. Cancer*, **59**, 527–534.

HOSPERS, G.A.P., MULDER, N.H., DE JONG, B., DE LEY, L., UGES, D.R.A., FICHTINGER-SCHEPMAN, A.M.J., SCHEPER, R.J. & DE VRIES, E.G.E. (1988). Characterization of a human small cell lung carcinoma cell line with acquired resistance to *cis*-Diamminedichloro platinum (II) *in vitro*. *Cancer Res.*, **48**, 6803–6807.

KELLAND, L.R., MURRER, B.A., ABEL, G., GIANDOMENICO, C.M., MISTRY, P. & HARRAP, K.R. (1992*a*). Ammine/amine platinum (IV) dicarboxylates: a novel class of platinum complex exhibiting selective cytotoxicity to intrinsically cisplatin-resistant human ovarian cell lines. *Cancer Res.*, **52**, 822–828.

KELLAND, L.R., JONES, M., ABEL, G. & HARRAP, K.R. (1992*b*). Human ovarian carcinoma cell lines and companion xenografts: a disease oriented approach to new platinum anticancer drug development. *Cancer Chemother. Pharmacol.*, **30**, 43–50.

KELLAND, L.R., MISTRY, P., ABEL, G., LOH, S.Y., O'NEILL, C.F., MURRER, B.A. & HARRAP, K.R. (1992*c*). Mechanism-related circumvention of cis-diamminedicholoroplatinum (II) acquired resistance using two pairs of human ovarian carcinoma cell lines by ammine/amine platinum (IV) dicarboxylates. *Cancer Res.*, **52**, 3857–3864.

MANGIONI, C., BOLIS, G., PECORELLI, S., BRAGMAN, K., EPIS, A., FAVALLI, G., GAMBINO, A., LANDONI, F., PRESTI, M., TORRI, W., VASSENA, L., ZANABONI, F. & MARSONI, S. (1989). Randomised trial in ovarian cancer comparing cisplatin and carboplatin. *J. Natl Cancer Inst.*, **81**, 1464–1468.

MCKEAGE, M.J., HIGGINS, J.D.(III). & KELLAND, L.R. (1991). Platinum and other metal coordination compounds in cancer chemotherapy. *Br. J. Cancer*, **64**, 788–792.

MISTRY, P., KELLAND, L.R., ABEL, G., SIDHAR, S. & HARRAP, K.R. (1991). The relationships between glutathione, glutathione-S-transferase and cytotoxicity of platinum drugs and melphalan in eight human ovarian carcinoma cell lines. *Br. J. Cancer*, **64**, 215–220.

OZOLS, R.F. (1991). Ovarian cancer: new clinical approaches. *Cancer Treat. Rev.*, **18** (suppl A), 77–83.

ROBERTS, J.J., KNOX, R.J., FRIEDLOS, F. & LYDALL, D.A. (1986). DNA as the target for the cytotoxic and anti-tumour action of platinum co-ordination complexes: comparative *in vitro* and *in vivo* studies of cisplatin and carboplatin. In McBrien, D.C.H. and Slater, T.F. (eds), *Biochemical Mechanisms of Platinum Antitumour Drugs*, pp. 29–64. Oxford: IRL Press.

ROSE, W.C. & BASLER, G.A. (1990). *In vivo* model development of cisplatin-resistant and -sensitive A2780 human ovarian carcinomas. *In Vivo*, **4**, 391–396.

SCHAPEL, F.M. Jr, SKIPPER, H.E., TRADER, M.W., LASTER, W.R. Jr, GRISWOLD, D.P. Jr & CORBETT, T.H. (1983). Establishment of cross-resistance profiles for new agents. *Cancer Treat. Rep.*, **67**, 905–922.

SEEBER, S., OSIEKA, R., SCHMIDT, C.G., ACHTERRATH, W. & CROOKE, S.T. (1982). *In vivo* resistance towards anthracyclines, etoposide and *cis*-diamminedichloro platinum (II). *Cancer Res.*, **42**, 4719–4725.

SESSA, C., VERMORKEN, J., RENARD, J., KAYE, S., SMITH, D., HUININK, TEN BOKKEL, W., CAVALLI, F. & PINEDO, H. (1988). Phase II study of iproplatin in advanced ovarian carcinoma. *J. Clin. Oncol.*, **6**, 98–105.

STEEL, G.G., COURTENAY, V.D. & PECKHAM, M.J. (1983). The response to chemotherapy of a variety of human tumour xenografts. *Br. J. Cancer*, **47**, 1–13.

TEICHER, B.A., HERMAN, T.S., HOLDEN, S.A., WANG, Y.M., PEEFFER, R., CRAWFORD, J.W. & FREI, E.III. (1990). Tumor resistance to alkylating agents conferred by mechanisms operative only *in vivo*. *Science*, **245**, 1457–1461.

WAUD, W.R., HARRISON, S.D. Jr, GILBERT, K.S., LASTER, W.R. Jr & GRISWOLD, D.P. Jr. (1991). Antitumor drug cross-resistance *in vivo* in a cisplatin-resistant murine P388 leukemia. *Cancer Chemother. Pharmacol.*, **27**, 456–463.

WEISS, G., GREEN, S., ALBERTS, D.S., THIGPEN, J.T., HINES, H.E., HANSON, K., PIERCE, H.I., BAKER, L.H. & GOODWIN, J.W. (1991). Second-line treatment of advanced measurable ovarian cancer with iproplatin: a Southwest Oncology Group study. *Eur. J. Cancer*, **27**, 135–138.

ZELLER, W.J., FRUHAUF, S., CHEN, G., KEPPLER, B.K., FREI, E. & KAUFMANN, M. (1991). Chemoresistance in rat ovarian tumours. *Eur. J. Cancer*, **27**, 62–67.